# Cardiovascular health and cancer risk associated with plant based diets: An umbrella review

**DOI:** 10.1371/journal.pone.0300711

**Published:** 2024-05-15

**Authors:** Angelo Capodici, Gabriele Mocciaro, Davide Gori, Matthew J. Landry, Alice Masini, Francesco Sanmarchi, Matteo Fiore, Angela Andrea Coa, Gisele Castagna, Christopher D. Gardner, Federica Guaraldi

**Affiliations:** 1 Department of Biomedical and Neuromotor Science, Alma Mater Studiorum–University of Bologna, Bologna, Italy; 2 Interdisciplinary Research Center for Health Science, Sant’Anna School of Advanced Studies, Pisa, Tuscany, Italy; 3 Department of Biochemistry, University of Cambridge, Cambridge, United Kingdom; 4 Stanford Prevention Research Center, Stanford University School of Medicine, Stanford, CA, United States of America; 5 Department of Translational Medicine, University of Eastern Piedmont, (UNIUPO), Novara, Italy; 6 IRCCS Istituto delle Scienze Neurologiche di Bologna, Programma Neurochirurgia Ipofisi—Pituitary Unit, Bologna, Italy; Federal University of Minas Gerais: Universidade Federal de Minas Gerais, BRAZIL

## Abstract

**Context:**

Cardiovascular diseases (CVDs) and cancer are the two main leading causes of death and disability worldwide. Suboptimal diet, poor in vegetables, fruits, legumes and whole grain, and rich in processed and red meat, refined grains, and added sugars, is a primary modifiable risk factor. Based on health, economic and ethical concerns, plant-based diets have progressively widespread worldwide.

**Objective:**

This umbrella review aims at assessing the impact of animal-free and animal-products-free diets (A/APFDs) on the risk factors associated with the development of cardiometabolic diseases, cancer and their related mortalities.

**Data sources:**

PubMed and Scopus were searched for reviews, systematic reviews, and meta-analyses published from 1st January 2000 to 31st June 2023, written in English and involving human subjects of all ages. Primary studies and reviews/meta-analyses based on interventional trials which used A/APFDs as a therapy for people with metabolic diseases were excluded.

**Data extraction:**

The umbrella review approach was applied for data extraction and analysis. The revised AMSTAR-R 11-item tool was applied to assess the quality of reviews/meta-analyses.

**Results:**

Overall, vegetarian and vegan diets are significantly associated with better lipid profile, glycemic control, body weight/BMI, inflammation, and lower risk of ischemic heart disease and cancer. Vegetarian diet is also associated with lower mortality from CVDs. On the other hand, no difference in the risk of developing gestational diabetes and hypertension were reported in pregnant women following vegetarian diets. Study quality was average. A key limitation is represented by the high heterogeneity of the study population in terms of sample size, demography, geographical origin, dietary patterns, and other lifestyle confounders.

**Conclusions:**

Plant-based diets appear beneficial in reducing cardiometabolic risk factors, as well as CVDs, cancer risk and mortality. However, caution should be paid before broadly suggesting the adoption of A/AFPDs since the strength-of-evidence of study results is significantly limited by the large study heterogeneity alongside the potential risks associated with potentially restrictive regimens.

## Introduction

Cardiovascular diseases (CVDs) and cancer currently represent the leading causes of death and disability worldwide. Studies performed on large cohorts worldwide have identified several modifiable and non-modifiable risk factors. Among them, robust evidence supports diet as a major modifiable risk factor [1].

A suboptimal diet, marked by insufficient consumption of fruits, vegetables, legumes, and whole grains, coupled with an excessive intake of meat (particularly red and processed), salt, refined grains and sugar, has been shown to notably elevate both mortality rates and disability-adjusted life years. Over time, these dietary choices have led to a concerning increase in health-related issues [1, 2].

Additionally, the reduction of products of animal origin in favor of vegetarian ones has been suggested to reduce CVD and cancer risk [3, 4]. Several major professional and scientific organizations encourage the adoption of vegetarian and vegan diets for the prevention and treatment of a range of chronic metabolic diseases such as atherosclerosis, type 2 diabetes, hypertension and obesity [5, 6]. Ethical, environmental, and socio-economic concerns have contributed to the widespread growth of plant-based diets, particularly vegetarian and vegan options [7–9]. 2014 cross-national governmental survey estimated that approximately 75 million people around the globe deliberately followed a vegetarian diet, while an additional 1,45 million were obliged to because of socio-economic factors [10, 11].

At the same time, study heterogeneity in terms of plant-based dietary regimens (from limitation of certain types to the total exclusion of animal products), their association with other lifestyle factors, patient demographic and geographical features, associated diseases, as well as study design and duration, significantly limit the assessment of the real benefits associated with animal-free and animal-products-free diets (A/APFDs). Finally, an increasing number of studies have highlighted the potential threatening consequences of chronic vitamin and mineral deficiencies induced by these diets (e.g., megaloblastic anemia due to vitamin B12 deficiency), especially more restrictive ones and in critical periods of life, like pregnancy and early childhood [5].

Based on these premises, our umbrella review aims at assessing the impact of animal-free and animal-products-free diets (A/APFDs) on the risk factors associated with the development of cardiometabolic diseases, cancer and their related mortalities in both the adult and the pediatric population, as well as pregnant women.

## Methods

### Search strategy

PubMed (https://pubmed.ncbi.nlm.nih.gov/) and Scopus (https://www.scopus.com/search/form.uri?display=basic#basic) databases were searched for reviews, systematic reviews and meta-analyses published from 1st January 2000 to 31st June 2023. We considered only articles written in English, involving human subjects, with an available abstract, and answering to the following PICO question: P (population): people of all ages; I (intervention) and C (comparison): people adopting A/APFDs vs. omnivores; O (outcome): impact of A/APFD on health parameters associated with CVDs, metabolic disorders or cancer.

Articles not specifying the type of A/APFD regimen were excluded. If not detailed, the A/APFDs adopted by study participants was defined as “mixed diet”. Vegetarian diets limiting but not completely excluding certain types of meat/fish (i.e. pesco- or pollo-vegetarian diet) were excluded. Studies focusing on subjects with specific nutritional needs (i.e., athletes or military personnel) -except pregnant women-, or with known underlying chronic diseases (i.e., chronic kidney disease), as well as articles focusing on conditions/health parameters related to disorders different from CVDs or cancer, and, finally, reviews/meta-analyses including interventional studies assessing A/APFDs comparing it with pharmacological interventions were excluded.

*Ad hoc* literature search strings, made of a broad selection of terms related to A/APFDs, including PubMed MeSH-terms, free-text words and their combinations, combined by proper Boolean operators, were created to search PubMed database: ((vegetari* OR vegan OR Diet, Vegetarian[MH] OR fruitar* OR veganism OR raw-food* OR lacto-veget* OR ovo-vege* OR semi-veget* OR plant-based diet* OR vegetable-based diet* OR fruit-based diet* OR root-based diet OR juice-based diet OR non-meat eate* OR non-meat diet*) AND ((review[Publication Type]) OR (meta-analysis[Publication Type]))) AND (("2000/01/01"[Date—Publication]: *"2023/06/31"[Date—Publication]))* and Scopus database: ALL(vegetari* OR vegan OR Diet, Vegetarian OR fruitar* OR veganism OR raw-food* OR lacto-veget* OR ovo-vege* OR semi-veget* OR plant-based diet* OR vegetable-based diet* OR fruit-based diet* OR root-based diet OR juice-based diet OR non-meat eate* OR non-meat diet) AND SUBJAREA(MEDI OR NURS OR VETE OR DENT OR HEAL OR MULT) PUBYEAR > 1999 AND (LIMIT-TO (DOCTYPE,*"re"))*

### Research design and study classification

An umbrella review approach [12] was applied to systematically assess the effect of A/APFDs on risk factors related to CVDs, metabolic disorders and cancer as derived from literature reviews, systematic reviews and meta-analyses (**Table 1**).

**Table 1:** List of included reviews.

*Title*	*Year*	*Main Area*	*Number of Included studies*	*R-AMSTAR rate*
Association between plant-based diets and plasma lipids: a systematic review and meta-analysis	2017	Lipids	19 RCT; 30 Cohort	0,95
Vegetarian diet, Seventh Day Adventists and risk of cardiovascular mortality: a systematic review and meta-analysis	2014	Cardiovascular	8 Cohort	0,86
The Relationship Between Plant-Based Diet and Risk of Digestive System Cancers: A Meta-Analysis Based on 3,059,009 Subjects	2022	Cancer	106 Case-Control; 82 Cohort	0,86
Risk of Incident Stroke among Vegetarians Compared to Nonvegetarians: A Systematic Review and Meta-Analysis of Prospective Cohort Studies	2021	Cardiovascular	7 Cohort	0,82
A systematic review and meta-analysis of changes in body weight in clinical trials of vegetarian diets	2015	Body Weight	8 RCT; 11 CT	0,82
Vegetarianism and breast, colorectal and prostate cancer risk: an overview and meta-analysis of cohort studies	2017	Cancer	9 Cohort	0,82
Effects of a vegetarian diet combined with aerobic exercise on glycemic control, insulin resistance, and body composition: a systematic review and meta-analysis	2023	Diabetes	9 RCT; 5 CT; 13 Cohort	0,82
Vegetarian, vegan diets and multiple health outcomes: A systematic review with meta-analysis of observational studies	2017	Cardiovascular, Lipids, Mortality, Cancer	86 Cross Sectional; 10 Cohort	0,79
Cardiometabolic risk factors in vegans; A meta-analysis of observational studies	2018	Cardiovascular	40 Cohort	0,79
Effects of Plant-Based Diets on Weight Status: A Systematic Review	2020	Body Weight	15 RCT; 4 CT	0,79
Animal versus plant-based protein and risk of cardiovascular disease and type 2 diabetes: a systematic review of randomized controlled trials and prospective cohort studies	2023	Cardiovascular, Diabetes	13 RCT; 7 Cohort	0,79
The effects of vegetarian diets on glycemia and lipid parameters in adult patients with overweight and obesity: a systematic review and meta-analysis	2023	Diabetes, Lipids	7 RCT	0,79
Effect of vegetarian dietary patterns on cardiometabolic risk factors in diabetes: A systematic review and meta-analysis of randomized controlled trials	2019	Cardiovascular	9 RCT	0,77
Vegetarian diets and blood pressure: a meta-analysis	2014	Cardiovascular	7 CT; 32 Cohort	0,77
Adherence to a Vegetarian Diet and Diabetes Risk: A Systematic Review and Meta-Analysis of Observational Studies	2017	Diabetes	12 Cross Sectional; 2 Cohort	0,77
Effect of vegetarian diets on the presentation of metabolic syndrome or its components: A systematic review and meta-analysis	2019	Cardiovascular, Lipids	6 RCT; 2 Cohort; 63 Cross Sectional	0,77
Vegetarian and vegan diets and the risk of cardiovascular disease, ischemic heart disease and stroke: a systematic review and meta-analysis of prospective cohort studies	2023	Cardiovascular	13 Cohort	0,77
Systematic review and meta-analysis of the associations of vegan and vegetarian diets with inflammatory biomarkers	2020	Cardiovascular	21 Cross Sectional	0,75
Effect of plant-based diets on obesity-related inflammatory profiles: a systematic review and meta-analysis of intervention trials	2016	Body Weight	23 RCT; 6 CT	0,75
Diets, Dietary Patterns, Single Foods and Pancreatic Cancer Risk: An Umbrella Review of Meta-Analyses	2022	Cancer	2 Cohort; 3 Case-Control	0,75
The effect of plant-based dietary patterns on blood pressure: a systematic review and meta-analysis of controlled intervention trials	2021	Cardiovascular	41 CT	0,72
Vegetarian-Based Dietary Patterns and their Relation with Inflammatory and Immune Biomarkers: A Systematic Review and Meta-Analysis	2019	Cardiovascular	7 RCT; 3 CT; 30 Cohort	0,72
Zinc Status of Vegetarians during Pregnancy: A Systematic Review of Observational Studies and Meta-Analysis of Zinc Intake	2015	Pregnancy	6 Cohort	0,7
Comparative effects of different dietary approaches on blood pressure in hypertensive and pre-hypertensive patients: A systematic review and network meta-analysis	2019	Cardiovascular	67 RCT	0,7
Comparison of plasma triacylglycerol levels in vegetarians and omnivores: a meta-analysis	2013	Lipids	6 Cohort; 6 Cross Sectional	0,7
Vegetarian Diets and Weight Reduction: a Meta-Analysis of Randomized Controlled Trials	2016	Body Weight	12 RCT	0,7
Association of vegetarian diet with inflammatory biomarkers: a systematic review and meta-analysis of observational studies	2017	Cardiovascular	18 Cross Sectional	0,7
Is a vegetarian diet safe to follow during pregnancy? A systematic review and meta-analysis of observational studies	2019	Pregnancy	19 Cohort	0,68
Effects of Vegetarian Diets on Blood Pressure Lowering: A Systematic Review with Meta-Analysis and Trial Sequential Analysis	2020	Cardiovascular	15 RCT	0,68
Association between Plant-Based Dietary Patterns and Risk of Cardiovascular Disease: A Systematic Review and Meta-Analysis of Prospective Cohort Studies	2021	Cardiovascular	10 Cohort	0,68
The Effect of Vegan Diets on Blood Pressure in Adults: A Meta-Analysis of Randomized Controlled Trials	2019	Cardiovascular	11 RCT	0,68
Dietary Patterns and Non-Communicable Disease Biomarkers: A Network Meta-Analysis and Nutritional Geometry Approach	2022	Lipids, Diabetes	59 RCT	0,68
Effects of Vegetarian Diets on Blood Lipids: A Systematic Review and Meta-Analysis of Randomized Controlled Trials	2015	Lipids	11 RCT	0,65
Comparison of vegetarian diets and omnivorous diets on plasma level of HDL-c: a meta-analysis	2014	Lipids	11 Cross Sectional; 1 Cohort	0,63
Systematic review of the impact of a plant-based diet on prostate cancer incidence and outcomes	2022	Cancer	5 RCT; 11 Cohort	0,63
Cardiovascular disease mortality and cancer incidence in vegetarians: a meta-analysis and systematic review	2012	Cardiovascular, Cancer	7 Cohort	0,61
Association of meat, vegetarian, pescatarian and fish-poultry diets with risk of 19 cancer sites and all cancer: findings from the UK Biobank prospective cohort study and meta-analysis	2022	Cancer	10 Cohort	0,55
Plant-Based Diet as a Strategy for Weight Control	2021	Body Weight	25 RCT; 2 Cohort	0,52
Effects of plant-based diets on plasma lipids	2009	Lipids	14 RCT; 10 Cross Sectional; 3 Case Control	0,52
Vegetarian Diet: A Prescription for High Blood Pressure? A Systematic Review of the Literature	2016	Cardiovascular	6 RCT; 1 CT; 32 Cross Sectional	0,47
Vegetarian diets in children: a systematic review	2017	Body Weight, Lipids	12 Cross Sectional; 12 Cohort	0,47
Plant-Based Diets and Lipid, Lipoprotein, and Inflammatory Biomarkers of Cardiovascular Disease: A Review of Observational and Interventional Studies	2022	Cardiovascular	31 RCT; 5 Cohort; 7 Cross Sectional	0,45
Plant-Based Diets and Cancer Risk: What is the Evidence?	2022	Cancer	2 RCT; 7 Cohort; 9 Case-Control; 1 Cross Sectional	0,36
Vegetarian diets and weight status	2006	Body Weight	8 RCT; 40 Observational	0,34
The effect of nutrition on stroke risk: A systematic review	2023	Cardiovascular	28 RCT	0,34
A comprehensive review of healthy effects of vegetarian diets	2023	Cardiovascular, Diabetes	59 RCT; 18 Cohort	0,34
A Comprehensive Review on the Effects of Vegetarian Diets on Coronary Heart Disease	2022	Cardiovascular	2 RCT; 3 Cohort; 1 Cross Sectional	0,31
Key elements of plant-based diets associated with reduced risk of metabolic syndrome	2014	Cardiovascular	1 CT; 3 Cross Sectional; 1 Cohort; 3 Case-Control	0,29

### Study selection

The list of articles identified by literature search was split into 5 equivalent parts, each assigned to a couple of readers (AC, DG, CW, ML, AM, FS, MF, AAC, GC and FG), who independently and blindly read the title and then the abstract of each article to define its pertinence. Papers included in the umbrella review had to focus on one/some of the following A/APFDs: vegans, lacto-vegetarians, ovo-vegetarians, lacto-ovo-vegetarians. No restriction was applied for age, gender, ethnicity, geographical origin, nor socio economic status. Primary studies, reviews/meta-analyses not written in English, or focusing on non-previously mentioned dietary regimens (including the Mediterranean diet) were excluded. Abstract meetings, editorials, letters to the editor, and study protocols were also excluded. To reduce study heterogeneity, at least in terms of dietary regimens, we excluded studies based on vegetarian regimens limiting but not avoiding fish or poultry, and prospective trials directly comparing A/AFPDs to pharmacological interventions.

**Fig 1:**
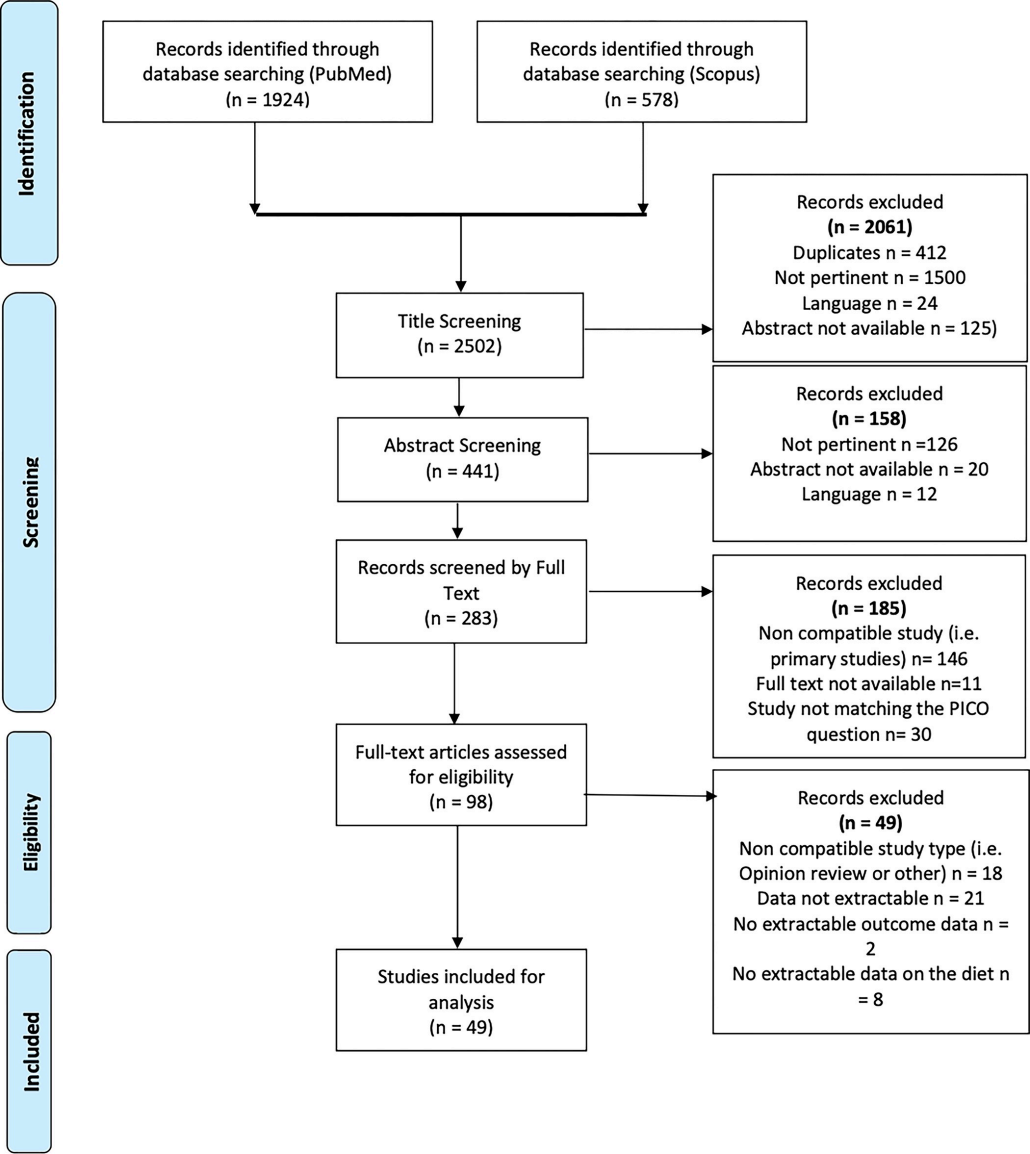
PRISMA flow-chart.

In case of discordance between readers, we resorted to discussion amongst the authors to resolve it, based on the article’s abstract or, if not decisive, the full text. The study selection process is summarized in **Fig 1**.

This review was registered on PROSPERO (Record ID: 372913 https://www.crd.york.ac.uk/prospero/display_record.php?RecordID=372913).

### Quality literature analysis

Three raters (AC, DG, FS) independently and blindly assessed the quality of the systematic reviews and meta-analyses using the revised AMSTAR-R 11-item tool, developed by the PEROSH group [13]. In case of disagreement, the score of each item and the final decision were discussed among the three raters.

### Data extraction and reporting

Ten investigators (AC, DG, GM, ML, AM, FS, MF, AAC, GC, FG) independently extracted data from eligible articles. Disagreements in data extraction were resolved by consensus. Using a predefined protocol and a Microsoft Excel sheet, the following data were extracted: first author’s affiliation country; type of review; type of diet; target population; number of aggregated participants; total cholesterol; HDL-cholesterol; LDL-cholesterol; triglycerides; apolipoprotein B; C-Reactive Protein (CRP); Body Mass Index (BMI); body weight; fasting glucose; glycosylated hemoglobin (HbA1c); systolic blood pressure; diastolic blood pressure; cardiac events (type; risk); cardiovascular diseases (type; risk); gestational diabetes; gestational hypertension; cancer (type; risk); death due to CVDs/cancer (risk). Data were reported as mean difference (MD), weighted mean difference (WMD), standardized mean difference (SMD), and 95%CI, while the estimated risk could be reported as relative risk (RR), odds ratio (OR), or hazard ratio (HR), according to the data reported by the study authors. Articles assessing the risk of gestational diabetes and hypertension, as well as risk of low birth weight, and their determinants were examined separately.

Results from studies focusing on both vegetarian and vegan diets were analyzed and reported separately if authors had stratified the results according to the type of diet. On the contrary, if data from vegan and vegetarian subjects were mixed, we arbitrarily considered all of them as “vegetarian”.

## Results

### Group 1: Cardiovascular endpoints and risk factors

#### I. Total cholesterol (TC)

Eight studies examined the levels of total serum cholesterol (TC) in vegetarians. Two focused on the general population and included 5,561 [14] and 576 [15] respectively, and, based on data meta-analysis, found a significant reduction in TC among vegetarians and people who assumed plant-based proteins (MD: -1.56 mmol/L; 95%CI: −1.73, −1.39; and -0.11 mmol/L; 95%CI: −0.22, −0.01, respectively).

Data were confirmed by Wang et al. (N = 832 total; Ovolacto/lacto-vegetarians: 291) [16], showing a greater dietary effect in subjects with a BMI ranging from 18.5 to 25 kg/m^2^ (mean TC reduction: −0.94 mmol/L; 95%CI: −1.33, −0.55), and from 25 to 30 kg/m^2^ (−0.58 mmol/L; 95%CI: −0.89, −0.27), than in those with a BMI >30 kg/m^2^ (−0.16 mmol/L; 95%CI: −0.30, −0.01), and by Xu et al. (N = 783) [17], reporting lower TC in overweight and obese people (WMD: −0.37 mmol/L; 95%CI: −0.52, −0.22) adopting a vegetarian diet.

Another systematic review by Elliott et al., including 27 randomized controlled trials on plant based vs. normal western diets [18], found lower TC levels in vegetarians. These results were in line with other two descriptive reviews, the first including 2,890 overweight/obese adults [19], the second 8,969 vegetarian children aged 0–18 years [20]. Furthermore, a meta-analysis by Liang et al. described significantly lower TC (from -0.36 to -0.24 mmol/L) in people adopting plant based diets vs. people adopting western habitual diets [21].

Moreover, the review and meta-analysis by Dinu et al. [14], based on 19 studies for a total of 1,272 adults, reported significantly lower levels of TC among vegans than in omnivores (WMD: −1.72 mmol/L; 95%CI: −1.93, −1.51).

#### II. High-density lipoprotein cholesterol (HDL-C)

Eight reviews focused on the effects of vegetarian diet on serum high-density lipoprotein cholesterol (HDL-C) levels. Six [15, 17, 18, 21–23] found no significant difference between vegetarians and omnivores, when considering normal weight and overweight/obese people. On the contrary, the study by Dinu et al. [14], based on 51 studies, for a total of 6,194 vegetarian adults, reported a WMD −0.15 mmol/L (95%CI: −0.19, −0.11). Liang et al. [21] analyzed 4 studies and reported a pooled estimated MD of −0.10 mmol/L (95%CI: −0.14, −0.05; p<0.001) in vegetarian diet adopters vs. western diets adopters. Finally, Zhang et al. [22] did not find any statistically significant differences in HDL-C levels when assessing vegetarian diets compared to non-vegetarians; on the same note Dinu et al. [14], analyzing data from 15 studies, for a total of 1,175 adults, found no significant differences in HDL-C levels between vegans and people following other dietary regimens.

#### III. Low-density lipoprotein cholesterol (LDL-C)

Ten reviews summarized the effect of vegetarian diets on serum levels of low-density lipoprotein cholesterol (LDL-C). Seven [14–18, 21, 23] found significantly lower LDL-C levels associated with vegetarian diet, both in the general population and in diabetic patients. In particular, Elliot et al. [18], analyzing 43 observational and interventional studies, described lower LDL-C in people adopting plant based diets; a significant difference was reported by the study of Liang et al. [21] based on 68 studies (MD: -0.29 to -0.17), and similar to data by Lamberg et al. [15], based on 13 RCTs including for a total of 576 participants (MD: -0.14 mmol/L; 95%CI: -0.25, -0.02). The impact of vegetarian diet appeared even greater in overweight or obese people, according to the analysis by Xu et al. [17], based on 7 RCTs (N = 783; MD: -0.31 mmol/L; 95%CI: -0.46, -0.16). Two reviews [19, 20] reported similar results in overweight/obese patients and children aged 0–18 years, but no meta-analyses were conducted. Wang et al. [16] reported a MD of −0.34 mmol/L (95%CI: −0.57, −0.11; p<0.001) in the general adult population. Ferdowsian et al. [23] reported an overall reduction of LDL-C associated with vegetarian diet, but no synthesis analyses were performed. Dinu et al. [14] analyzed 46 studies encompassing 5,583 vegetarians and found a WMD of -1.18 mmol/L (95%CI: -1.34, -1.01). Finally, Viguiliouk et al. [24] reported a MD of −0.12 mmol/L (95%CI: −0.20, −0.04) in 6 trials involving 602 diabetic patients.

Four reviews identified a significant reduction in LDL-C in vegans as compared to omnivores [14, 19, 23, 25]. Benatar et al. [25] analyzed 31 studies, for a total of 3,355 healthy vegan adults and 53,393 non-vegan controls and found MD of -0.49 mmol/L (95%CI: -0.62, -0.36; p<0.0001). Ferdowsian et al. [23] reported a reduction of LDL-C in healthy vegans, and Ivanova et al. [19] in overweight patients, but no meta-analysis was performed. Finally, Dinu et al. [14] analyzed 13 studies, for a total of 728 healthy vegan adults, and found a significant LDL-C reduction (WMD: −1.27 mmol/L; 95%CI: −1.66, −0.88).

#### IV. Triglycerides (TG)

Seven systematic reviews [14, 16–18, 20, 23, 26] analyzed serum triglycerides (TG) in vegetarians vs. omnivores. Specifically, Wang et al. [16] described no differences between the two, with a pooled estimated effect of 0.04 mmol/L (95%CI: −0.05, 0.13; p *=* 0.4). Zhang et al. [26] analyzing 12 studies for a total of 1,300 subjects, found a MD of −1.28 mmol/L (95%CI; −2.14, −0.42). Schürmann et al. and Ferdowsian et al. [20, 23] reported lower TG in vegetarians in both children and adults but did not perform data meta-analysis. Dinu et al. [14] analyzed 55 studies including 4,008 vegetarians and found a WMD of −0.63 mmol/L (95%CI: −0.97, −0.30; p = 0.02). Conversely, in the review by Elliott et al. [18] no differences were reported in triglycerides. Xu et al. [17] reported a significant increase of TG (WMD: 0.29 mmol/L; 95%CI: 0.11, 0.47) in vegetarians as compared to meat eaters.

The effect of vegan diet on TG remains debated as one review [23] reported significative changes in TGs (-0.14 mmol/L, CI -0.24 to -0.05), while another [14] did not find any differences between vegans and omnivores since, after having analyzed 13 studies for 483 vegans, they reported a WMD of -0.52 mmol/L (95%CI: -1.13; 0.09).

#### V. C-reactive protein (CRP)

Three studies reported lower C-reactive protein (CRP) levels in normal weight, overweight and obese vegetarians as compared to non-vegetarians. Craddock et al. and Menzel et al. reported a WMD of -0.61 mg/L (95%CI: -0.91, -0.32; p = 0.0001) [27]; -0.25 mg/L (95%CI: -0.49, 0; p = 0.05) [28], respectively.

Data derived from the analysis by Menzel et al. [28] in vegan subjects were in line with previously mentioned studies performed in vegetarians (WMD: -0.54 mg/L; 95%CI: -0.79, -0.28; p<0.0001).

Two reviews [29, 30] focused on the effects of mixed vegetarian diets on CRP levels. The first [29] included 2,689 obese patients and found a WMD of -0.55 mg/L (95%CI: -0.78, -0.32; I^2^ = 94.4%), while the other [30], based on 2,398 normal weight subjects found no significant differences between vegetarians and omnivores in the primary analysis; alas, when considering a minimum duration of two years vegetarianism they described lower CRP levels vs. omnivores (Hedges’ g = -0.29; 95%CI: -0.59, 0.01).

#### VI. Plant-based diets and lipids

Three studies [23, 26, 31] assessed the lipid profile in people following plant-based diets (without differentiating among diet subtypes) in comparison with omnivores. All of them found significantly lower levels of TC, HDL-C and LDL-C in subjects following plant-based diets. Specifically, Yokoyama et al. [31] reported a WMD of −1.62 mmol/L (95%CI: −1.92, −1.32; p< 0.001; I^2^ = 81.4) for TC, −1.27 mmol/L (95%CI: −1.55, −0.99; p< 0.001; I^2^ = 83.3) for LDL-C, −0.2 mmol/L (95%CI: −0.26, −0.14; p< 0.001; I^2^ = 49.7) for HDL-C, and −0.36 mmol/L; 95%CI: −0.78, 0.06; p = 0.092; I^2^ = 83.0) for TG when considering observational studies, and of −0.69 mmol/L (95%CI: −0.99, −0.4; p<0.001; I^2^ = 54.8) for TC, −0.69 mmol/L (95%CI: −0.98, −0.37; p<0.001; I^2^ = 79.2) for LDL-C, −0.19 mmol/L (95%CI: −0.24, −0.14; p<0.001; I^2^ = 8.5) for HDL-C, and a non-statistically significant increase of TG based on prospective cohort studies. Additionally, Zhang et al. [26] in their meta-analysis, including 1,300 subjects, found a SMD of -1.28 mmol/L in TG (95% CI -2.14 to -0.42).

Finally, Picasso et al. [32] did not find any differences in triglycerides for mixed vegetarian diets (MD: 0.04 mmol/L; 95%CI: -0.09, 0.28), but did find statistically significant differences in HDL-C (MD: -0.05 mmol/L; 95%CI: -0.07, -0.03).

#### VII. Blood pressure

*A*. *Systolic blood pressure (SBP)*. Various studies found significantly lower mean levels of systolic blood pressure (SBP) levels in vegetarians compared to the general population [33–36]. Specifically, Gibbs et al. [33] reported a SMD of -5.47 mmHg (95%CI: -7.60, -3.34; p<0.00001) in ovo-lacto-vegetarians, as did Lee et al. [34] reporting a SMD of -1.75 mmHg (95%CI: -5.38, 1.88; p = 0.05); furthermore, they reported a SBP decreased by -2.66 mmHg (95%CI: -3.76, -1.55), in people adopting generic vegetarian diets. Moreover, Garbett et al. [35] reported a 33% lower prevalence of hypertension in vegetarians vs. nonvegetarians. On the contrary, Schwingshackl et al. [36], analyzing data from 67 clinical trials overall including 17,230 pre-hypertensive and hypertensive adult patients with a BMI between 23.6 and 45.4 kg/m^2^, followed for 3 to 48 months, did not find any significant reductions in SBP associated with vegetarian diet.

Four reviews investigated the differences in SBP between vegans and non-vegans. Benatar et al. and Lee et al. [25, 34] reported significantly lower mean SBP levels in vegans vs. omnivores (MD: -2.56 mmHg; 95%CI: -4.66, -0.45; and WMD: -3.12 mmHg; 95%CI: -4.54, -1.70; p<0.001, respectively). On the other hand, Gibbs et al. [-1.30 mmHg (95%CI: -3.90,1.29)] and Lopez et al. (-1.33 mmHg; 95%CI: −3.50, 0.84; *P* = 0.230) [33, 37] did not find any significant difference in mean SBP levels between vegans and omnivores.

Both reviews [32, 38] focusing on SBP in mixed-plant-based dietary patterns found significantly lower levels in vegetarians than in omnivores. The meta-analysis by Picasso et al. [32], based on 4 RCTs did not find any differences, alas, analyzing 42 cross sectional studies, they described a MD of -4.18 mmHg (95%CI -5.57, -2.80; p<0.00001), in agreement with Yokoyama et al. [38], who reported a MD of -4.8 mmHg (95%CI: -6.6, -3.1; p<0.001; I^2^ = 0) according to the 7 controlled trials, 6 of which being randomized (311 participants), included in the analysis, and of -6.9 mmHg (95%CI: -9.1, -4.7; p<0.001; I^2^ = 91.4) based on the other 32 observational studies (21,604 participants).

*B*. *Diastolic blood pressure (DBP)*. Garbett et al. [35] reported reduced mean diastolic blood pressure (DBP) values in vegetarians vs. omnivores, confirmed by the analysis of Gibbs et al. [33] (WMD: –2.49 mmHg; 95%CI: –4.17, –0.80; p = 0.004; I^2^ = 0%) in ovo-lacto-vegetarians, by Lee et al. [34] [WMD: -1.69 mmHg (95%CI: -2.97, -0.41; p<0.001)] who included 15 randomized controlled trials (N = 856) performed in vegetarians; and by Yokoyama et al. [38], who highlighted a MD -2.2 mmHg (95%CI: -3.5, -1.0; p<0.001; I^2^ = 0%) and -4.7 mmHg (95%CI: -6.3, -3.1; p<0.001; I^2^ = 92.6%) according to data from 7 controlled trials (N = 311) and 32 observational studies (N = 21,604), respectively. Conversely, Schwingshackl et al. [36] did not find significant differences between vegetarians and non-vegetarians.

Three reviews [25, 34, 37] examined the impact of vegan vs. non-vegan diet on DBP and described statistically significant reductions. Benatar et al. described reduction of DBP, corresponding to a MD of -1.33 mmHg (95%CI: -2.67, -0.02) [25]. Lee et al. described a reduction in DBP of a WMD of -1.92 mmHg (95%CI: -3.18, -0.66; p<0.001) [34]. Finally, Lopez et al. [37] described the same reduction amounting to WMD: -4.10 mmHg (95%CI: -8.14, -0.06).

Four studies agreed upon the lower mean DBP levels in subjects following mixed vegetarian diets as compared to omnivores [32–34, 38], quantified as MD -3.03 mmHg (95%CI: -4.93, 1.13; p = 0.002) by Picasso et al. [32], and −2.2 mmHg (95%CI: −3.5, −1.0; p<0.001) and −4.7 mmHg (95%CI: −6.3, −3.1; p <0.001) by the analysis performed on clinical trials and observational studies, respectively, by Yokoyama et al. [38].

#### VIII. Body weight and body mass index (BMI)

Berkow et al. [39] identified 40 observational studies comparing weight status of vegetarians vs. non-vegetarians: 29 reported that weight/BMI of vegetarians of both genders, different ethnicities (i.e., African Americans, Nigerians, Caucasians and Asians), and from widely separated geographic areas, was significantly lower than that of non-vegetarians, while the other 11 did not find significant differences between the two groups. In female vegetarians, weight was 2.9 to 10.6 kg (6% to 17%) and BMI 2.7% to 15.0% lower than female non-vegetarians, while the weight of male vegetarians was 4.6 to 12.6 kg (8% to 17%) lower and the BMI 4.6% to 16.3% lower than that of male non-vegetarians. The review by Schürmann et al. [20], focusing on 8,969 children aged 0–18 years old found similar body weight in both vegetarian and vegan children as compared to omnivore ones. Dinu et al. [14] analyzed data from 71 studies (including 57,724 vegetarians and 199,230 omnivores) and identified a WMD BMI of -1.49 kg/m^2^ (95%CI: -1,72, -1,25; p<0.0001) in vegetarians when compared to omnivores.

Barnard et al. [40] found a significant reduction in weight in pure ovolactovegetarians (−2.9 kg; 95% CI −4.1 to −1.6; P<0.0001), compared to non-vegetarians from control groups; furthermore, they found in vegans the mean effect was of -3.2 kg (95% CI: -4.0;-2.4, P: <0.0001); overall they included 490 subjects in their analysis, excluding subjects who did not complete the trials.

Benatar et al. [25]–including 12,619 vegans and 179,630 omnivores from 40 observation studies–and Dinu et al. [14]–based on 19 cross sectional studies, for a total of 8,376 vegans and 123,292 omnivores–reported the same exact result, with a mean lower BMI in vegans vs omnivores, equal to -1.72 kg/m^2^ (95%CI: -2.30, -1.16) and -1.72 kg/m^2^ (95%CI: -2.21,-1.22; p<0.0001), respectively. The meta-analysis by Long et al. [41], performed on 27 studies, reported a MD of -0.70 kg/m^2^ (95%CI: -1.38, -0.01) for BMI in vegans vs. omnivores. A systematic review and meta-analysis by Agnoli et al. [42] found mean BMI to be lower in subjects adhering to mixed vegetarian diets as compared to omnivores. Additionally, Tran et al. [43] described weight reductions in clinically healthy patients, as well as in people who underwent vegetarian diets as a prescription, but no meta-analysis was performed.

Finally, Huang et al. [44] found significant differences in both vegans and vegetarians, who were found to have lost weight after having adopted the diet as a consequence of being assigned to the intervention group in their randomized studies. For vegetarians the WMD was -2.02 kg (95%CI: -2.80 to -1.23), when compared to mixed diets, and for vegans the WMD was -2.52 kg (95%CI: -3.02 to -1.98), when compared to vegetarians.

#### IX. Glucose metabolism

Viguiliouk et al. [24] found a significant reduction in HbA1c (MD: −0.29%; 95%CI: −0.45, −0.12) and fasting glucose (MD: −0.56 mmol/L; 95%CI: −0.99, −0.13) in vegetarians vs. non-vegetarians.

The meta-analysis by Dinu et al. [14], reported for vegetarians (2256) vs omnivores (2192) WMD: -0.28 mmol/L (95%CI: -0.33, -0.23) in fasting blood glucose.

These findings were confirmed by Picasso et al. [32] who found a MD of -0.26 mmol/L (95% CI: -0.35, -0.17) in fasting glucose in mixed-vegetarian diets as compared to omnivores.

A meta-analysis by Long et al. [41], based of 27 cross sectional studies, showed a MD for homeostasis model assessment of insulin resistance -measured as HOMA-IR, a unitless measure ideally less than one- of -0.75 (95%CI: -1.08, -0.42), fasting plasma glucose in vegetarians who adhered also to an exercise intervention as compared to omnivores.

Lee & Park [45] reported a significantly lower diabetes risk (OR 0.73; 95%CI: 0.61, 0.87; p<0.001) in vegetarians vs. non-vegetarians, being the association stronger in studies conducted in the Western Pacific region and Europe/North America than in those from Southeast Asia.

Regarding vegans, the review by Benatar et al. [25] determined a mean reduction of 0.23 mmol/L (95%CI: -0.35, -0.10) of fasting blood glucose in vegans (N = 12,619) as compared to omnivores (N = 179,630). The finding was in line with Dinu et al. [14], who reported a WMD of -0.35 mmol/L (95%CI: -0.69, -0.02; p = 0.04) of fasting blood glucose in vegans (n = 83) than omnivores (n = 125).

A systematic review, finally, including 61 studies [42] found mean values of fasting plasma glucose, and T2D risk to be lower in subjects following mixed vegetarian diets as compared to omnivores.

#### X. Cardiovascular events

Huang et al. [46] found a significantly lower risk of ischemic heart disease (IHD) (RR: 0.71; 95%CI: 0.56, 0.87), but no significant differences for cerebrovascular mortality between vegetarians and non-vegetarians. The review by Remde et al. [47] was not conclusive, as only a few studies showed a reduction of the risk of CVDs for vegetarians versus omnivores, while the others did not find any significant results.

Dybvik et al. [48] based on 13 cohort studies for a total of 844,175 participants (115,392 with CVDs, 30,377 with IHD and 14,419 with stroke) showed that the overall RR for vegetarians vs. nonvegetarians was 0.85 (95%CI: 0.79–0.92, I^2^ = 68%; 8 studies) for CVD, 0.79 (95%CI: 0.71–0.88, I^2^ = 67%; 8 studies) for IHD, 0.90 (95%CI: 0.77–1.05, I^2^ = 61%; 12 studies) for total stroke, while the RR of IHD in vegans vs. omnivores was 0.82 (95%CI: 0.68–1.00, I^2^ = 0%; 6 studies).

The meta-analysis by Kwok et al. [49], based on 8 studies including 183,321 subjects comparing vegetarians versus non-vegetarians. They identified a significant reduction of IHD in the Seventh Day Adventist (SDA) cohort, who primarily follow ovo-lacto-vegetarian diets, while other non-SDA vegetarian diets were associated only with a modest reduction of IHD risk, raising the concern that other lifestyle factors typical of SDA and, thus not generalizable to other groups, play a primary role on outcomes. IHD was significantly reduced in both genders (RR: 0.60; 95%CI: 0.43, 0.83), while the risk of death and cerebrovascular disease and cardiovascular mortality risk reduction was significantly reduced only in men. No significant differences were detected for the risk of cerebrovascular events.

The meta-analysis by Lu et al. [50] -657,433 participants from cohort studies- reported a lower incidence of total stroke among vegetarians vs. nonvegetarians (HR = 0.66; 95%CI = 0.45–0.95; I^2^ = 54%), while no differences were identified for incident stroke.

The descriptive systematic review by Babalola et al. [3] reported that adherence to a plant-based diet was inversely related to heart failure risk and advantageous for the secondary prevention of CHD, particularly if started from adolescence. Another review by Agnoli et al. [42], confirmed a lower incidence of CVDs associated with mixed vegetarian diets as compared to omnivorous diets. Finally, Chhabra et al. [51] found that vegetarian diet, particularly if started in adolescence and associated with vitamin B intake, can reduce the risk of stroke.

Gan et al. [52] described a lower risk of CVDs (RR 0.84; 95% CI 0.79 to 0.89; *p* < 0.05) in high, vs. low, adherence plant based diets, but the same association was not confirmed for stroke (RR 0.87; 95% CI: 0.73, 1.03).

### Group 2: Pregnancy outcomes

The meta-analysis by Foster et al. [53], performed on 6 observational studies, found significantly lower zinc levels in vegetarians than in meat eaters (-1.53 ± 0.44 mg/day; p = 0.001), but no association with pregnancy outcomes, specifically no increase in low children birth weight. The finding was confirmed by Tan et al. [54], who similarly reported no specific risks, but reported that Asian (India/Nepal) vegetarian mothers exhibited increased risks to deliver a baby with Low Birth Weight (RR: 1.33 [95%CI:1.01, 1.76, p =  0.04, I^2^ = 0%]; nonetheless, the WMD of neonatal birth weight in five studies they analyzed suggested no difference between vegetarians and omnivores.

To our knowledge, no reviews/meta-analyses have assessed the risk of zinc deficiency and its association with functional outcomes in pregnancy in relation to mixed or vegan diets.

### Group 3: Cancer

The meta-analysis by Parra-Soto et al. [55], based on 409,110 participants from the UK Biobank study (mean follow-up 10.6 years), found a lower risk of liver, pancreatic, lung, prostate, bladder, colorectal, melanoma, kidney, non-Hodgkin lymphoma and lymphatic cancer as well as overall cancer (HR ranging from 0.29 to 0.70) determined by non-adjusted models in vegetarians vs. omnivores; when adjusted for sociodemographic and lifestyle factors, multimorbidity and BMI, the associations remained statistically significant only for prostate cancer (HR 0.57; 95%CI: 0.43, 0.76), colorectal cancer (HR 0.73; 95%CI: 0.54, 0.99), and all cancers combined (HR 0.87; 95%CI 0.79, 0.96). When colorectal cancer was stratified according to subtypes, a lower risk was observed for colon (HR 0.69; 95%CI: 0.48, 0.99) and proximal colon (HR 0.43; 95%CI: 0.22, 0.82), but not for rectal or distal cancer.

Similarly, the analysis by Huang et al. [46], based on 7 studies for a total of 124,706 subjects, reported a significantly lower overall/total cancer incidence in vegetarians than non-vegetarians (RR 0.82; 95%CI: 0.67, 0.97).

Zhao et al. [56] found a lower risk of digestive system cancer in plant-based dieters (RR = 0.82, 95%CI: 0.78–0.86; p< 0.001) and in vegans (RR: 0.80; 95%CI: 0.74, 0.86; p<0.001) as compared to meat eaters.

Additionally, DeClercq et al. [57] reported a decreased risk of overall cancer and colorectal cancer, but inconsistent results for prostate cancer and breast cancer; this was substantiated by Godos et al. [58] found no significant differences in breast, colorectal, and prostate cancer risk between vegetarians and non-vegetarians.

The umbrella review by Gianfredi et al. [59], did describe a lower risk of pancreatic cancer associated with vegetarian diets.

Dinu et al. [14] reported a reduction in the risk of total cancer of 8% in vegetarians, and of 15% in vegans, as compared to omnivores. They described lower risk of cancer among vegetarians (RR 0.92; 95%CI 0.87, 0.98) and vegans (RR: 0.85; 95%CI: 0.75,0.95); nonetheless, they also described non-significant reduced risk of mortality from colorectal, breast, lung and prostate cancers. Regarding the latter, a meta-analysis by Gupta et al. [60] on prostate cancer risk found a decreased hazard ratio for the incidence of prostate cancer (HR: 0.69; 95%CI: 0.54–0.89, P<0.001) in vegetarians as compared to omnivores from the evidence coming from 3 studies. In the vegan population, similar results were observed from the only included study (HR: 0.65; 95%CI: 0.49–0.85; p<0.001).

### Group 4: Death by cardiometabolic diseases and cancer

According to Huang et al. [46], the mortality from IHD (RR: 0.71; 95%CI: 0.56, 0.87), circulatory diseases (RR: 0.84; 95%CI: 0.54, 1.14) and cerebrovascular diseases (RR: 0.88; 95%CI: 0.70, 1.06) was significantly lower in vegetarians than in non-vegetarians.

The analysis by Dinu et al. [14] performed on 7 prospective studies, overall including 65,058 vegetarians, reported a 25% reduced mortality risk from ischemic heart diseases (RR 0.75; 95%CI: 0.68, 0.82; p<0.001), but no significant differences were found analyzing 5 cohort studies in terms of mortality from CVDs, cerebrovascular diseases, nor colorectal, breast, prostate, and lung cancer. Regarding vegans, they analyzed 6 cohort studies, and found no differences in all-cause mortality, but significant differences in cancer incidence (RR: 0.85; 95%CI: 0.75, 0.95), indicating a protective effect of vegan diets.

The literature search did not identify studies focusing on mortality risk for cardiometabolic and cancer diseases in vegans.

### Quality of the included studies

The quality of the 48 reviews and meta-analyses included in this umbrella review was assessed through the AMSTAR-R tool. Results are reported in **S1 Table**. Overall, the average quality score was 28, corresponding to mean quality. However, 36 studies (75%) scored between 60% and 90% of the maximum obtainable score, and can, therefore, be considered of good/very good quality. The least satisfied item on the R-AMSTAR grid was #8 -scientific quality of included studies used to draw conclusions-, where as many as 19 studies (39.6%) failed to indicate the use of study-related quality analysis to make recommendations. This finding should be read in conjunction with the missing quality analysis in 15 studies (31.3%)–Item #7 scientific quality of included studies assessed and documented-. Item #10, regarding publication bias, was the second least met item, in which 18 studies (37.5%) did not perform any analysis on this type of bias. 16 studies (33.3%) lacked to indicate careful exclusion of duplicates (Item #2), but also the presence of conflict of interest (Item #11). This point is certainly another important piece to consider in the overall quality assessment of these articles. All these considerations give us a picture of a general low quality of the publications found, lowering the strength of evidence as well as the external validity of the results.

## Discussion

This umbrella review provides an update on the benefits associated with the adoption of A/AFPDs in reducing risk factors associated with the development of cardiometabolic diseases and cancer, considering both the adult and the pediatric population, as well as pregnant women.

Compared to omnivorous regimens, vegetarian and vegan diets appear to significantly improve the metabolic profile through the reduction of total and LDL cholesterol [14–21, 23, 25], fasting blood glucose and HbA1c [14, 24, 25, 37, 39–41], and are associated with lower body weight/BMI, as well as reduced levels of inflammation (evaluated by serum CRP levels [27, 30]), while the effect on HDL cholesterol and triglycerides, systolic and diastolic blood pressure levels remains debated. A much more limited body of literature suggested vegetarian, but not vegan diets also reduce ApoB levels further improving the lipid profile [61].

It should be remarked that, in the majority of the cases, people adopting plant-based diets are more prone to engage in healthy lifestyles that include regular physical activity, reduction/avoidance of sugar-sweetened beverages, alcohol and tobacco, that, in association with previously mentioned modification of diet [62], lead to the reduction of the risk of ischemic heart disease and related mortality, and, to a lesser extent, of other CVDs.

The adoption of vegan diets is known to increase the risk of vitamin B-12 deficiency and consequent disorders–for which appropriate supplementation was recommended by a 2016 position paper of the Academy of Nutrition and Dietetics’ [5], but, apparently, does not modify the risk of pregnancy-induced hypertension nor gestational diabetes mellitus [53, 54].

The three meta-analyses [46, 55, 57] that analyzed the overall risk of cancer incidence in any form concordantly showed a reduction in risk in vegetarians compared to omnivores. These general results were inconsistent in the stratified analyses for cancer types, which as expected involved smaller numbers of events and wider confidence intervals, especially for less prevalent types of cancers.

The stratified analyses in the different reviews did not show any significant difference for bladder, melanoma, kidney, lymphoma, liver, lung, or breast cancer. Conversely the three meta-analyses that addressed colorectal cancer [55, 57, 58] showed a decrease in risk in two out of three with one not showing a significant difference in vegetarians versus omnivores for the generic colorectal tract.

Interestingly, one review [55] showed how analysis with even more specific granularity could reveal significant differences in particular subsets of cancers, e.g., distal, and proximal colon. Also, another recent review found significant results for pancreatic cancer [59].

Our umbrella review seems consistent with other primary evidence that links the consumption of red processed meats to an increased risk of cancers of the gastro-intestinal tract [63]. The association certainly has two faces, because while a potential risk of cancer given by increased red meat consumption can be observed, the potential protective factor given by increased fruit and vegetable consumption, shown by other previous evidence, must also be considered [64].

It has also been described that vegetarians, in addition to reduced meat intake, ate less refined grains, added fats, sweets, snacks foods, and caloric beverages than did nonvegetarians and had increased consumption of a wide variety of plant foods [65]. Such a dietary pattern seems responsible for a reduction of hyperinsulinemia, one of the possible factors for colorectal cancer risk related to diet and food intake [66, 67]. In the same manner, some research has suggested that insulin-like growth factors and its binding proteins may relate to cancer risk [68, 69]. This dietary pattern should not be regarded as a universal principle, as varying tendencies have been observed among vegetarians and vegans in different studies. This pattern of consumption may potentially negate the anticipated beneficial effects of their diets.

Also, some protective patterns can be attributed to the effects of bioactive compounds of plant foods, these being primary sources of fiber, carotenoids, vitamins, minerals, and other compounds that have been associated with anti-cancer properties [70, 71]. The protective patterns are likely attributed to the mechanistic actions of the many bioactives found in plant foods such as fiber, carotenoids, vitamins, and minerals with plausible anti-cancer properties. These ranged from epigenetic mechanisms [72], to immunoregulation, antioxidant and anti-inflammatory activity [73, 74].

Finally, increased adiposity could be another pathway by which food intake is associated with these types of cancers. Since our umbrella review has demonstrated that vegetarian diets are associated with lower BMI, this might be another concurrent factor in the decreased risk for pancreatic and colorectal cancers in vegetarians.

Inflammatory biomarkers and adiposity play pivotal roles in the genesis of prostate cancer [75, 76], hence the same etiological pathways might be hypothesized even for the increase of this type of cancer in people adopting an omnivorous diet.

The study presents several noteworthy strengths in its methodological approach and thematic focus. It has employed a rigorous and comprehensive search strategy involving two major databases, PubMed, and Scopus, spanning over two decades of research from 1^st^ January 2000 to 31^st^ June 2023, thereby ensuring a robust and exhaustive collection of pertinent literature. By utilizing an umbrella review, the research enables the synthesis of existing systematic reviews and meta-analyses, providing a higher level of evidence and summarizing a vast quantity of information. Furthermore, its alignment with current health concerns, specifically targeting cardiovascular diseases and cancer, makes the study highly relevant to ongoing public health challenges and positions it as a valuable resource for informing preventive measures and dietary guidelines. The deployment of blinded and independent assessments by multiple raters and investigators fortifies the research by minimizing bias and reinforcing the reliability of the selection, quality assessment, and data extraction processes. Quality assessment is standardized using the revised AMSTAR-R 11-item tool, and transparency is fostered through registration on PROSPERO, thus enhancing the credibility of the study. Lastly, the study’s detailed analysis and reporting, particularly the extraction of specific health measures such as cholesterol levels, glucose levels, blood pressure, and cancer risks, contribute to the comprehensiveness of the data synthesis, thereby underlining the overall integrity and significance of the research.

Main limitations to data analysis and interpretation are intrinsic to the original studies and consist in the wide heterogeneity in terms of sample size, demographic features, and geographical origin of included subjects, dietary patterns–not only in terms of quality, but, even more important and often neglected, quantity, distribution during the day, processing, cooking methods–and adherence, and other lifestyle confounders. In this regard, it is worth to mention that the impact of diet *per se* on the development of complex disorders (i.e. CVDs and cancer) and related mortality is extremely difficult to assess [71], especially in large populations, characterized by a highly heterogeneous lifestyle. It should also be considered the heterogeneity in dietary and lifestyle habits among countries, according to which the adoption of A/AFPDs could modify significantly habits in some countries, but not in others, and consequently have an extremely different impact on the risk of developing cardiometabolic disorders and cancer [25]. Furthermore, due to the nature of umbrella reviews, the present work may not include novel associations which were excluded from the analyzed reviews, as the main aim was to summarize secondary studies, such as reviews and meta-analyses. Finally, studies assessing the benefit of A/AFPDs on cancer risk are also limited by the heterogeneity in the timing of oncological evaluation and, therefore, disease progression, as well as in the histological subtypes and previous/concomitant treatments [72–75].

## Conclusions

In conclusion, this umbrella review offers valuable insights on the estimated reduction of risk factors for cardiometabolic diseases and cancer, and the CVDs-associated mortality, offered by the adoption of plant-based diets through pleiotropic mechanisms. Through the improvement of glycolipid profile, reduction of body weight/BMI, blood pressure, and systemic inflammation, A/AFPDs significantly reduce the risk of ischemic heart disease, gastrointestinal and prostate cancer, as well as related mortality.

However, data should be taken with caution because of the important methodological limitation associated with the original studies. Moreover, potential risks associated with insufficient intake of vitamin and other elements due to unbalanced and/or extremely restricted dietary regimens, together with specific patient needs should be considered, while promoting research on new and more specific markers (i.e. biochemical, genetic, epigenetic markers; microbiota profile) recently associated with cardiometabolic and cancer risk, before suggesting A/AFPDs on large scale.

## Supporting information

S1 Table R-AMSTAR. (XLSX)

S2 Table PRISMA 2020 checklist. (PDF)
